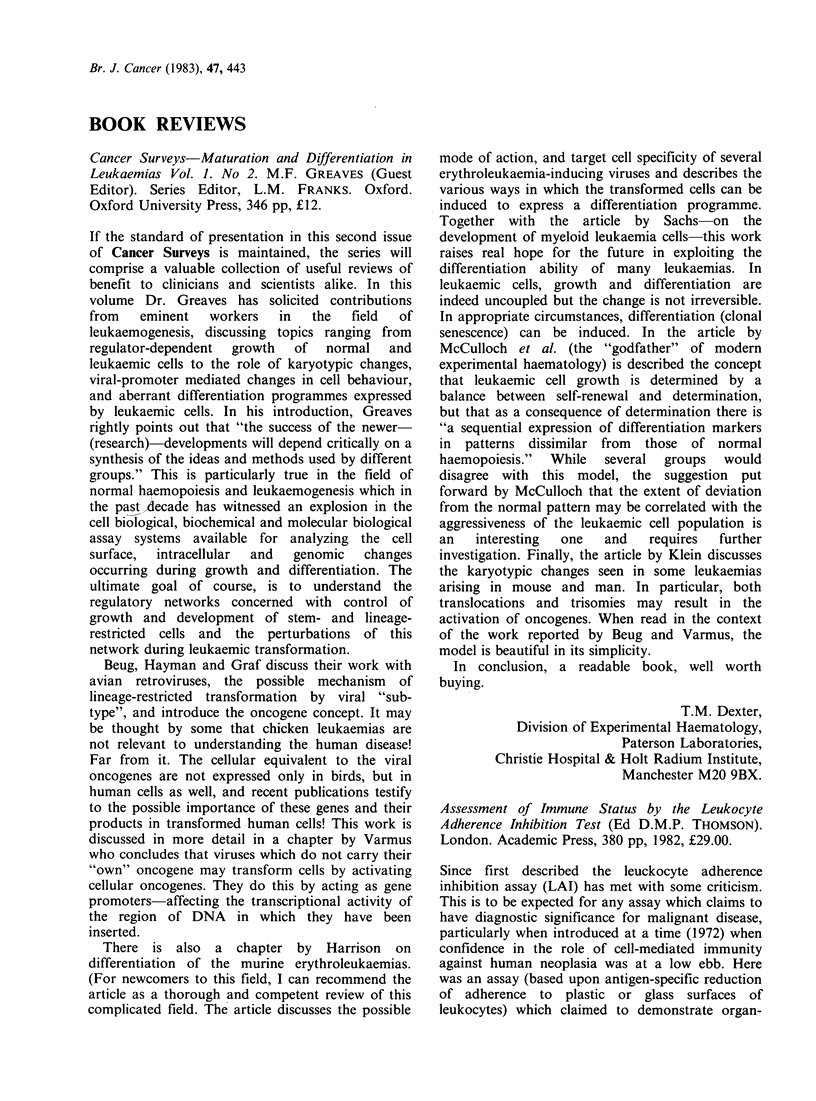# Cancer Surveys—Maturation and Differentiation in Leukaemias Vol. 1. No 2

**Published:** 1983-03

**Authors:** T.M. Dexter


*Br. J. Cancer* (1983), **47**, 443

### BOOK REVIEWS

*Cancer Surveys—Maturation and Differentiation in Leukaemias Vol. 1. No 2.* M.F. GREAVES (Guest Editor). Series Editor, L.M. FRANKS. Oxford. Oxford University Press, 346 pp, £12.

If the standard of presentation in this second issue of **Cancer Surveys** is maintained, the series will comprise a valuable collection of useful reviews of benefit to clinicians and scientists alike. In this volume Dr. Greaves has solicited contributions from eminent workers in the field of leukaemogenesis, discussing topics ranging from regulator-dependent growth of normal and leukaemic cells to the role of karyotypic changes, viral-promoter mediated changes in cell behaviour, and aberrant differentiation programmes expressed by leukaemic cells. In his introduction, Greaves rightly points out that "the success of the newer—(research)—developments will depend critically on a synthesis of the ideas and methods used by different groups." This is particularly true in the field of normal haemopoiesis and leukaemogenesis which in the past decade has witnessed an explosion in the cell biological, biochemical and molecular biological assay systems available for analyzing the cell surface, intracellular and genomic changes occurring during growth and differentiation. The ultimate goal of course, is to understand the regulatory networks concerned with control of growth and development of stem- and lineage-restricted cells and the perturbations of this network during leukaemic transformation.

Beug, Hayman and Graf discuss their work with avian retroviruses, the possible mechanism of lineage-restricted transformation by viral "sub-type", and introduce the oncogene concept. It may be thought by some that chicken leukaemias are not relevant to understanding the human disease! Far from it. The cellular equivalent to the viral oncogenes are not expressed only in birds, but in human cells as well, and recent publications testify to the possible importance of these genes and their products in transformed human cells! This work is discussed in more detail in a chapter by Varmus who concludes that viruses which do not carry their "own" oncogene may transform cells by activating cellular oncogenes. They do this by acting as gene promoters—affecting the transcriptional activity of the region of DNA in which they have been inserted.

There is also a chapter by Harrison on differentiation of the murine erythroleukaemias. (For newcomers to this field, I can recommend the article as a thorough and competent review of this complicated field. The article discusses the possible mode of action, and target cell specificity of several erythroleukaemia-inducing viruses and describes the various ways in which the transformed cells can be induced to express a differentiation programme. Together with the article by Sachs—on the development of myeloid leukaemia cells—this work raises real hope for the future in exploiting the differentiation ability of many leukaemias. In leukaemic cells, growth and differentiation are indeed uncoupled but the change is not irreversible. In appropriate circumstances, differentiation (clonal senescence) can be induced. In the article by McCulloch *et al.* (the "godfather" of modern experimental haematology) is described the concept that leukaemic cell growth is determined by a balance between self-renewal and determination, but that as a consequence of determination there is "a sequential expression of differentiation markers in patterns dissimilar from those of normal haemopoiesis." While several groups would disagree with this model, the suggestion put forward by McCulloch that the extent of deviation from the normal pattern may be correlated with the aggressiveness of the leukaemic cell population is an interesting one and requires further investigation. Finally, the article by Klein discusses the karyotypic changes seen in some leukaemias arising in mouse and man. In particular, both translocations and trisomies may result in the activation of oncogenes. When read in the context of the work reported by Beug and Varmus, the model is beautiful in its simplicity.

In conclusion, a readable book, well worth buying.

T.M. Dexter,
Division of Experimental Haematology,
Paterson Laboratories,
Christie Hospital & Holt Radium Institute,
Manchester M20 9BX.